# Advances in microbial mevalonolactone production: from fermentative mevalonate accumulation to downstream lactonization

**DOI:** 10.1016/j.engmic.2025.100257

**Published:** 2025-12-24

**Authors:** Hao Tang, Lihong Yin, Yiwen Jiang, Teng Xu, Ting Xue, Meimei Fu, Jianghao Chen, Jinshan Guo

**Affiliations:** a Suzhou Institute of Biomedical Engineering and Technology, Chinese Academy of Science, Suzhou, 215011, China; b Hangzhou Ji Bei Technology Co., Ltd, Hangzhou 311121, China

**Keywords:** Mevalonolactone, Microbial cell factories, Biosynthesis, Metabolic engineering, Mevalonate lactonization

## Abstract

Mevalonolactone (MVAL) is a high-value feedstock for the cosmetic industry, with (*R*)-(-)-MVAL as the sole bioactive enantiomer. Chemical synthesis, which is the traditional method for MVAL production, is hindered by cumbersome procedures, low chiral purity, and sensitivity to humidity. Microbial fermentation *via* fermentative mevalonate (MVA) accumulation followed by *in vitro* acid-catalyzed lactonization has emerged as a promising alternative for producing optically pure (*R*)-(-)-MVAL. Strategies for MVA overproduction in microbial systems are reviewed, including the selection of chassis strains and enzymes for the MVA biosynthetic pathway, metabolic engineering approaches for strain improvement, optimization of fermentation processes, and downstream processes for MVA-to-MVAL lactonization. Finally, prospects for advancing microbial MVAL production are discussed.

## Graphical abstract

**Figure:**
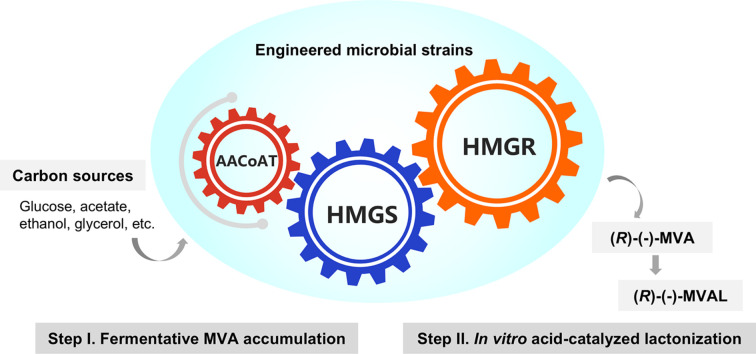

## 1 Introduction

**Fig. 1:**
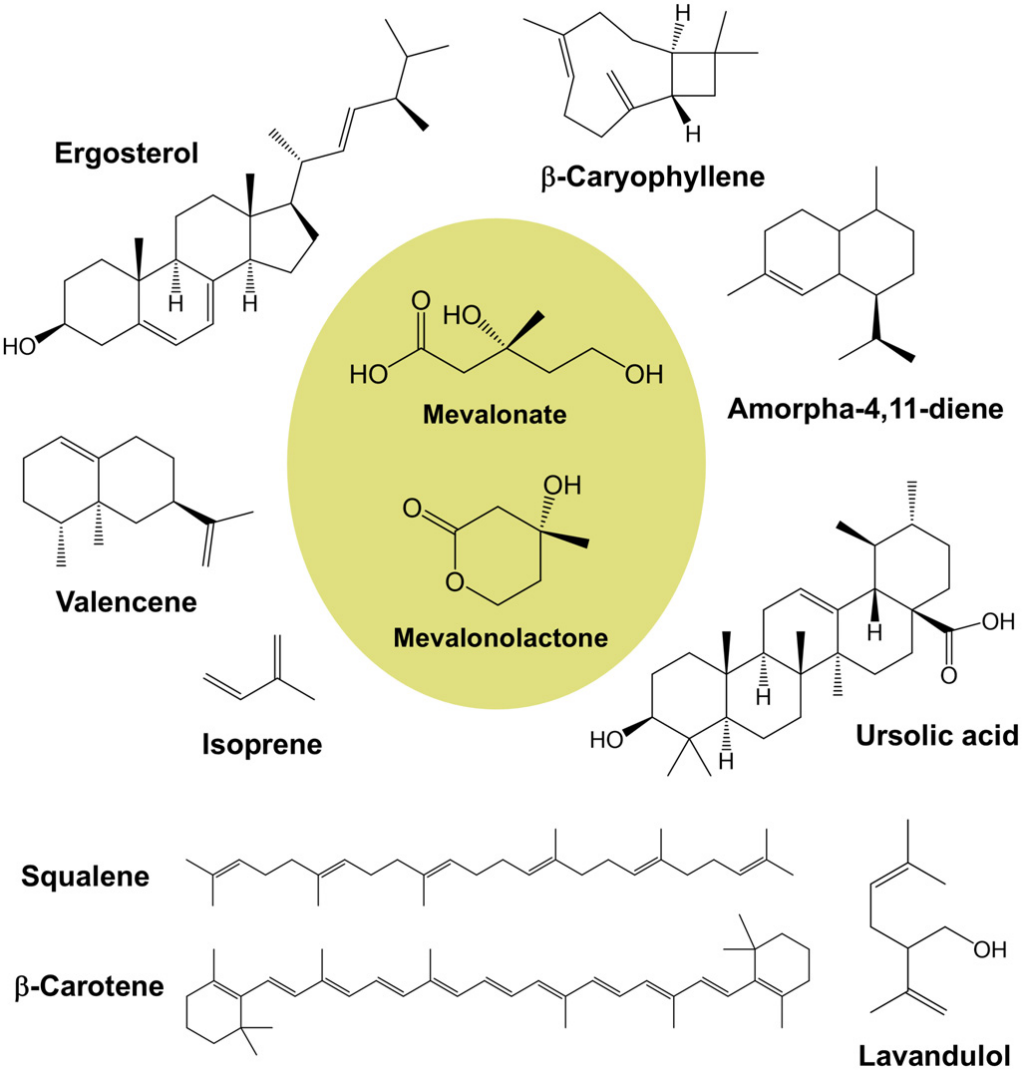
MVA, MVAL, and representative naturally occurring downstream compounds of the MVA pathway.

**Fig. 2:**
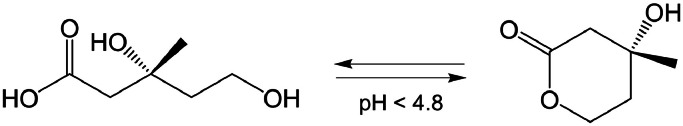
Mutual transformation of MVA (left) and MVAL (right).

Mevalonate (MVA) is a key intermediate in a broad spectrum of cellular biological processes and regulations. Its significance as a biosynthetic precursor of terpenes, vitamins, and sterols has been previously demonstrated (Fig. 1) [1]. Mevalonolactone (MVAL), a cyclic lactone, is a commercially preferred industrial feedstock over MVA. In 1956, Folkers *et al.* isolated and elucidated its structure [2]. As shown in Fig. 2, MVA can be converted to MVAL *via* intramolecular dehydration condensation between its carboxylic acid and terminal hydroxyl groups in acidic aqueous environments (pH < 4.8), enabling the reversible interconversion of MVA and MVAL [3]. Notably, only the (*R*)-(-)-enantiomer of MVAL exhibits biological activity, reflecting the stereoselectivity of biological systems that exclusively utilize this chiral form in biochemical reactions and metabolic processes [4]. MVAL has gained widespread application in the cosmetics, food, and other industries [5]. In skincare, it facilitates the functional restoration of the damaged stratum corneum and possesses excellent anti-aging and anti-wrinkle effects. As a superior humectant, its unique hydrophilic structure can form a cross-linked network film on the skin surface to lock in moisture, delivering a smooth yet non-greasy texture with remarkable moisturizing performance [6]. These properties make it a critical component of various skincare products, including creams, whitening lotions, and serums. Owing to these versatile applications, MVAL synthesis has garnered significant research interest. Concurrently, its market demand has surged with the global expansion of biotechnology, underscoring broad industrial prospects [7].

**Fig. 3:**
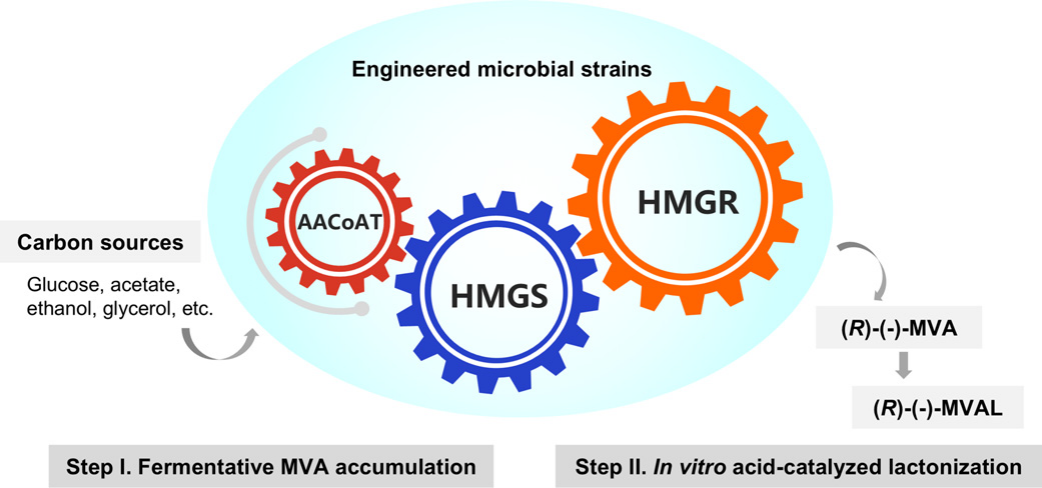
Schematic diagram of fermentative MVA accumulation and downstream *in vitro* conversion of MVA to MVAL. Abbreviations: AACoAT, acetyl-CoA acetyltransferase; HMGS, 3-hydroxy-3-methylglutaryl-CoA synthase; HMGR, 3-hydroxy-3-methylglutaryl-CoA reductase.

Currently, chemical synthesis remains the dominant method of MVAL production by domestic manufacturers. This traditional route uses 4-chloro-2-butanone as the raw material and involves multiple steps, including esterification, the Reformatsky reaction, hydrolysis, and cyclization [8]. However, it suffers from inherent drawbacks: the process is cumbersome with a low overall yield, and each step requires precise regulation of the reaction parameters, such as temperature, pressure, and the presence of a catalyst, to ensure product purity and yield. Additionally, the final product is typically a racemic mixture with an equimolar ratio of *R*- and *S*-configurations. Improving the chiral purity requires complex purification steps, which significantly increases production costs [9]. Our experimental observations, which were conducted by consecutively replicating the methods described previously at the laboratory scale in Guangzhou, China (a humid location) and Changchun, China (an arid region) [8,10,11], further revealed that the product yield of chemically synthesized MVAL was significantly impaired in humid climates (unpublished data). In humid areas, additional air dehumidification equipment is required, which introduces operational inconveniences and increases costs. In contrast, microbial fermentation offers substantial advantages, particularly chiral synthesis and cost control. ADEKA Corporation (Japan; formerly Asahi Denka Co., Ltd.) pioneered the large-scale microbial fermentation production of naturally occurring *R*-configured MVAL with optical activity; this product has been commercialized at a price of approximately 2 million yen per kilogram. In comparison, the racemic mixture still costs 4 million yen per kg, highlighting the significant cost advantages of microbial fermentation. At present, the mainstream biosynthetic strategy for MVAL comprises two sequential steps: first, accumulating MVA *via* microbial fermentation, and then converting MVA to MVAL through acid-catalyzed *in vitro* lactonization (Fig. 3). Notably, the direct microbial synthesis of MVAL remains unachieved. Various microbial strains have been widely used as chassis for MVA synthesis, including *Saccharomycopsis fibuligera* (employed by ADEKA), *Yarrowia lipolytica, Saccharomyces cerevisiae, Escherichia coli, Pseudomonas putida*, and *Methylobacterium extorquens* (Table 1).

**Table 1:** Studies showing increased MVA production by microorganisms.

Host strain	Strategy	Fermentation scale	MVA titer (g/L)	Reference
*S. fibuligera*				
IFO 0107	1) Addition of Triton X-100. 2) Utilization of organic nitrogen sources. 3) Precision carbon feeding based on pH or DO.	20-L bioreactor	12.1	[18]
IFO 0107	Replacement of costly malt extract and yeast extract with an inexpensive combination of casein-derived peptone and corn steep liquor as organic nitrogen sources.	20-L bioreactor	14.1	[21]
IFO 0107	Pressure screening of IFO 0107 with the HMGR competitive inhibitor ML-236B.	20-L bioreactor	19.4	[19]
*S. cerevisiae*				
CEN.PK2-1C	CRISPR/Cas9-mediated knockouts of *rox1* (a repressor inhibiting MVA pathway genes), *bts1* (a branch-point enzyme in isoprenoid biosynthesis), *ypl062w*, and *yjl064w* (uncharacterized genes enhancing MVA flux), together with the truncation of the *erg9* promoter.	Shake Flask	Not reported	[22]
BY4741	1) Deletion of the mitochondrial *IDH1* gene to shunt citrate from the TCA cycle to the cytoplasm. 2) Overexpression of *A. nidulans* ACL for citrate-to-acetyl-CoA conversion. 3) Introduction of *E. faecalis* MVA pathway genes. 4) Replacement of the ERG12 promoter with the copper-regulated P*_CTR3_* to block downstream metabolism.	Shake flask	0.03	[23]
CAR-0002	ERG10, HMGS and tHMGR were fused with the P3:GCN:P4 coiled-coil domain to form enzyme complexes, thereby boosting directed metabolic flux.	Shake flask	Not reported	[16]
CEN.PK2-1C	1) Integration of MVA pathway genes from *E. faecalis* and the *Se-acs*^L641P^ gene (encoding ACS) from *S. enterica* into the *S. cerevisiae* genome. 2) Knockout of the *ADH1* and *GPD1* genes. 3) δ-integration-based replacement of plasmids for the genomic integration of *mvaE*/*mvaS*/*Se-acs*^L641P^. 4) Metabolic flux diversion was minimized by regulating ERG9. 5) Acetyl-CoA biosynthesis reinforcement by *CAB1* overexpression and pantothenate supplementation.	2-L bioreactor	13.3 ± 0.5	[15]
*Y. lipolytica*				
ZG03	1) Downregulation of *ERG12 via* promoter replacement. 2) Overexpression and copy number amplification of *HMGR* and *ERG13*. 3) Enhancement of acetyl-CoA supply *via* overexpression of the mitochondrial citrate transporter YHM2, cytosolic citrate lyases ACL1/2, and deletion of mitochondrial isocitrate dehydrogenases (IDH1/2). 4) Further introduction of a high-activity citrate lyase AnACLa/b from *A. nidulans*. 5) Deletion of the citrate efflux protein CEX1.	2-L bioreactor	101	[17]
*E. coli*				
XL1-Blue	Introduction of *mvaE* and *mvaS* from *E. faecalis*.	2-L bioreactor	47	[24]
DH10B	1) Overexpression of the *MevT* operon (comprising *E. coli atoB* and *S. cerevisiae HMGS/tHMGR*). 2) Additional copy number increase of *tHMGR* for enhanced HMG-CoA-to-MVA flux and elimination of toxic HMG-CoA accumulation.	Shake flask	4.2	[57]
BL21star^TM^ (DE3)	1) Introduction of *mvaE* and *mvaS* from *E. faecalis*. 2) Mutation of MvaS (A110G) to enhance enzymatic activity.	Shake flask	3.1	[53]
BW25113	Introduction of *mvaE* and *mvaS* from *L. casei*.	1.3-L bioreactor	88	[26]
BW25113	1) Chromosomal integrated *atoB, mvaS*, and *mvaE* genes from *L. casei*, driven by promoter M1-93, replacing the native *adhE* gene (encoding alcohol dehydrogenase) to eliminate by-product formation. 2) Deletion of *atpFH* (encoding the b and c subunits of the H^+^-ATP synthase) to enhance glycolytic flux and accelerate glucose consumption. 3) Integration of an extra copy of the *atoB*-*mvaS*-*mvaE* operon to strengthen downstream flux from acetyl-CoA to MVA, with concurrent replacement of the native *ldhA* gene (encoding lactate dehydrogenase). 4) Knockout of *sucA* (encoding the E1 subunit of 2-oxoglutarate decarboxylase) to block the TCA cycle, redirecting acetyl-CoA toward MVA synthesis. 5) Optimization of the amount of yeast extract added in the culture medium.	Shake flask	30	[61]
MG1655	Introduction of *mvaE* and *mvaS* from *E. faecalis*.	2.5-L bioreactor	65	[9]
BL21 (DE3)	1) Co-expression of *acs* from *E. coli* W3110 as well as *mvaE* and *mvaS* from *E. faecalis*. 2) Two-stage fermentation strategy of glucose for biomass accumulation and feed switching to acetate accompanied by IPTG induction for expression.	5-L bioreactor	7.85	[66]
BW25113	1) Deletion of the chromosomal *pgi* gene (encoding phosphoglucose isomerase). 2) Expression of *pgi* under the control of the T5 promoter and the *Lac* operon. 3) Tuning EMP pathway flux by adjusting IPTG induction levels to avoid excessive acetyl-CoA shunting from an overly high EMP flux. 4) Introduction of *mvaE* and *mvaS* from *E. faecalis* in plasmid form, under the control of the constitutive promoter P*_trp_*.	Shake flask	Not reported	[68]
BW25113	1) Design and construction of the EP-bifido pathway, including: heterologous introduction of *B. adolescentis fxpk* (encoding phosphoketolase), knockout of *pfkA* in the BW25113 chassis, and overexpression of *E. coli fbp* (encoding fructose-1,6-bisphosphatase). 2) Introduction of *atoB* from *E. coli* MG1655, and *mvaS*/*mvaA* from *L. casei*.	Shake flask	11.2	[83]
MG1655	1) Introduction of the *mvaE* and *mvaS* genes from *E. faecalis*. 2) Overexpression of the endogenous *atoB* gene. 3) The *gltA* gene (encoding citrate synthase) was disrupted to strengthen intracellular acetyl-CoA availability. 4) Cell surface display of BGL with direct conversion of cellobiose to MVA.	Test tube	5.7	[67]
BW-P	1) Replacement of the promoter of *zwf* (encoding glucose-6-phosphate dehydrogenase) for enhanced PPP flux to boost NADPH supply. 2) CRISPRi-based downregulation of *pfkA* with dynamic control of EMP flux and avoidance of growth inhibition.	Shake flask	11.2	[71]
DH5α	1) Utilization of glycerol as a fermentation carbon source. 2) Introduction of the *mvaES* operon from *E. faecalis*. 3) Enhancement of acetyl-CoA supply *via* assembly of an xPK-PTA bypass to enhance acetyl-CoA supply. 4) Overexpression of 6-phosphogluconate dehydrogenase to optimize NADPH regeneration.	Shake flask	7.21	[56]
BL21 (DE3)	The *mvaE* and *mvaS* genes from *E. faecalis* were fused with RBDs *via* the 0DPP7 RNA scaffold system to achieve spatial co-aggregation of the MVA enzymes.	Shake flask	3.13	[65]
BL21 (DE3)	1) Expression control of *E. faecalis mvaE via* the Tet promoter (aTc-inducible) and the *E. faecalis mvaS via* T7 promoter (IPTG-inducible). 2) Acetate utilization as the carbon source. 3) Precise repression of *gltA via* the McbR-mediated control of the *gltA* promoter to redirect partial carbon flux to MVA. 4) Repeated fed-batch fermentation in shake flasks for addressing rapid acetate consumption *via* continuous acetate feeding.	Shake flask	7.9	[64]
*P. putida*				
KT2440	1) Introduction of *atoB* from *E. coli* and *mvaE*/*mvaS* from *E. faecalis*. 2) Utilization of ethanol as the carbon source. 3) Knockout of *endA* (encoding endonuclease I) and *endX* (encoding extracellular DNA endonuclease) to stabilize MVA gene expression by preventing the degradation of exogenous plasmids. 4) Knockout of *qedH-I* and *qedH-II* (encoding quinoprotein ethanol dehydrogenase) and expression of *E. coli acs* to reduce the ethanol oxidation rate to avoid acetate surge and the acceleration of acetate conversion to acetyl-CoA. 5) Optimization of pH in batch fermentation.	2.5-L bioreactor	4.6	[75]
KT2440	1) Introduction of *mvaE* and *mvaS* from *E. faecalis*. 2) CRISPRi-based inhibition of *glpR*, with alleviation of transcriptional inhibition of *glpFKRD* (its products are responsible for glycerol uptake and catabolism).	Shake flask	0.237	[58]
KT2440	1) Introduction of *mvaE* and *mvaS* from *E. faecalis*. 2) Overexpression of the endogenous *atoB* gene. 3) Utilization of 2,3-BDO as the carbon source. 4) Optimization of aeration parameter (shaking flask speed) to balance growth and MVA production.	Shake flask	2.21	[59]
KT2440	1) Genomic integration of CRISPRa system components dCas9/MCP-SoxS. 2) Driving of the *mvaES* operon from *E. faecalis* by the CRISPRa-responsive promoter J3-BBa_J23117. 3) Activation of *mvaES* transcription *via* J306 scaffold RNA targeting the J3-BBa_J23117 promoter.	Shake flask	0.402 ± 0.021	[60]
GZT23	1) Introduction of *atoB, mvaS*, and *mvaE* driven by one J23111 promoter. 2) Regulation of their expression *via* RBSs of different strengths. 3) Further DO-stat fed-batch fermentation strategy (maintaining DO at around 20% *via* automatic low-concentration glucose feeding upon glucose depletion, stabilizing the pH at 7.0 by adding flow-through NH_4_OH, with an initial glucose concentration of 20 g/L).	5-L bioreactor	5	[29]
*M. extorquens*				
AM1	1) Introduction of *hmgcs1* (encoding a high-activity HMGS) from *B. germanica* and *tchmgr* (encoding a high-activity HMGR) from *T. cruzi*. 2) Introduction of *phaA* (encoding acetyl-CoA acetyltransferase) from *R. eutropha* to enhance precursor supply. 3) Further tuning of the RBS strength upstream of *phaA* to balance pathway flux.	5-L bioreactor	2.22	[31]
AM1	1) Construction of the AraC-mev-based MVA biosensor Sensor-4 and its integration at the *celABC* locus. 2) Integration of the RBS-optimized MVA synthesis pathway Mvt-3 (comprising *hmgcs1, tchmgr*, and *phaA*) at the *attTn7* locus. 3) Random mutagenesis of the QscR, screening to identify the mutant QscR-49 regulator, and its overexpression to redirect carbon flux toward acetyl-CoA.	5-L bioreactor	2.67	[30]

**Fig. 4:**
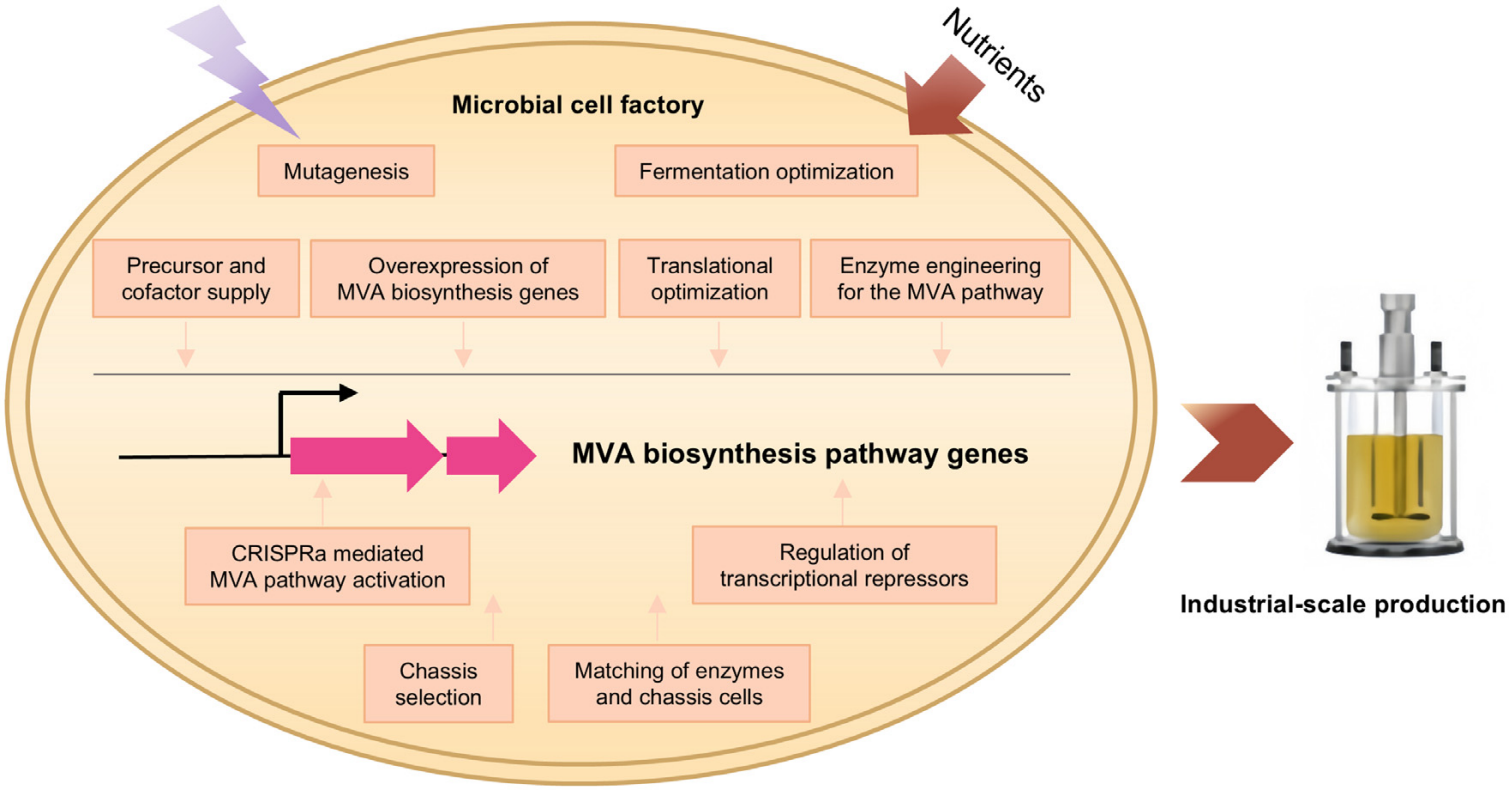
Strategies for MVA biosynthesis in a microbial chassis. Efforts, including chassis selection, matching of enzymes and chassis cells, increasing precursor and cofactor supply, overexpression of MVA biosynthesis genes, translational optimization, enzyme engineering for the MVA pathway, mutagenesis, fermentation process optimization, CRISPRa mediated MVA pathway activation, and regulation of transcriptional repressors, were employed for the production of MVA in microbial cell factories.

This review provides an overview of microbial MVAL bioproduction, focusing on the key aspects of MVA biosynthesis, including chassis strain selection, screening of high-activity MVA pathway enzymes, and diverse metabolic engineering strategies for strain enhancement, such as the overexpression of MVA pathway structural genes, modulation of acetyl-CoA precursor and cofactor availability, transcriptional regulator engineering, fermentation process optimization (Fig. 4), and downstream lactonization for converting MVA to MVAL.

## 2 Chassis strains for MVA production

### 2.1 Yeasts

Yeasts are widely exploited as microbial chassis for the biosynthesis of high-value compounds, with *S. cerevisiae* and *Y. lipolytica* being the most intensively studied because of their desirable biological characteristics and excellent engineering feasibility [[12], [13], [14]]. MVA is a naturally occurring metabolite ubiquitous in yeasts [15]. However, its natural titer varies significantly among different yeast species and strains, with some exhibiting relatively high basal production and others very low. For strains with very low basal production levels, such as *S. cerevisiae* CAR-0002 [16] and *Y. lipolytica* ZG03 [17], metabolic engineering of the yeast chassis is required to boost MVA biosynthesis. For *S. fibuligera* strains, the overproducing mutants currently used for industrial MVA production are derived through classical screening and selection methods [18], or *via* targeted mutagenesis approaches such as resistance-based screening [19]. Notably, the genetic engineering of MVA biosynthetic pathways has not yet been applied to *S. fibuligera*.

In 1968, Tamura *et al*. [20] systematically screened 1,006 type strains (including yeasts, fungi, actinomycetes, and bacteria) obtained from the Institute of Applied Microbiology, University of Tokyo. Among these isolates, *Endomycopsis fibuliger* IAM 4347 (later reclassified as *S. fibuligera* NRRL Y-9069) exhibited the highest yield and most consistent production, with a titer of 939 mg/L [18,20]. ADEKA Corporation pioneered the large-scale microbial fermentation of MVA using *S. fibuligera* IFO 0107 and achieved an MVA titer of 14.1 g/L after 12 days of cultivation in a 20-L fermenter [21]. Additionally, two variant strains derived from *S. fibuligera* IFO 0107, ADK 8107, and ADK 8108, achieved titers of 19–19.4 g/L under the same cultivation scale and duration [19]. Building on these advances, ADEKA subsequently realized the well-established industrialization of MVAL. Among all the microbial chassis reported to date, *Y. lipolytica* has the highest MVA titer. Zhang *et al*. [17] demonstrated that engineered *Y. lipolytica* produced 101 g/L of MVA in a 1.3-L bioreactor, which remains the only documented study on MVA biosynthesis using *Y. lipolytica* as a chassis. Meanwhile, the highest reported titer for *S. cerevisiae* was 13.3 g/L, which was achieved using strain CEN.PK2-1C in one study [15]. Other studies on *S. cerevisiae*-based MVA production either did not specify the titer [16,22] or reported considerably lower yields [23].

### 2.2 Bacteria

Prokaryotes that inherently possess the MVA pathway (e.g., *Enterococcus faecalis*) are not suitable for efficient MVA synthesis because of their slow growth rates, weak acetyl-CoA supply capacity, and lack of mature gene-editing tools [24]. In contrast, three commonly used prokaryotic hosts, *E. coli, P. putida*, and *M. extorquens* naturally lack a complete MVA biosynthetic pathway and thus do not produce MVA endogenously.

Given its highly mature genetic manipulation tools and distinct advantages, such as a short fermentation cycle [13,25], *E. coli* has become the preferred chassis for heterologous MVA production, resulting in the most extensive research reports in this field. Furthermore, *E. coli* cannot utilize MVA as a carbon source, which inherently facilitates accumulation of the target metabolite. For instance, Tabata *et al*. [24] reported in 2004 that the *E. coli* chassis strain XL1-Blue exhibited excellent production performance: an MVA titer of 47 g/L was achieved within 50 h in a 2-L fed-batch fermenter. This work is the first to demonstrate that *E. coli* can serve as a high-quality host for MVA production and remains a foundational study in the field of *E. coli* metabolic engineering for MVA biosynthesis. Notably, among the current studies on MVA production using *E. coli* chassis, strain BW25113 exhibited the most impressive performance, achieving a titer of 88 g/L in a 1.3-L bioreactor [26]. Other *E. coli* strains typically yield MVA titers ranging from several grams per liter to several tens of grams per liter (e.g., XL1-Blue, MG1655) (Table 1). Two key factors can be proposed to account for this phenomenon. First, *E. coli* BW25113 benefits from optimal compatibility with heterologous enzymes. Unlike other *E. coli* chassis strains that rely primarily on the *E. faecalis mvaE*/*mvaS* operon, BW25113 was engineered to express the *L. casei mvaS*/*mvaE* module. As validated *via* systematic enzyme screening [26], this enzyme combination demonstrated superior compatibility with the BW25113 chassis (see Section 3.1.1). Second, this superiority is attributed to the inherent metabolic advantages of the BW25113 chassis itself, particularly the naturally abundant supply of the acetyl-CoA precursor [27]. Overall, *E. coli* chassis demonstrated excellent performance for MVA production and hold significant potential for industrial applications.

As for *P. putida*, which is widely recognized as a highly capable microbial chassis, it exhibits excellent environmental tolerance and broad utilization of low-cost carbon sources [13,28]. Regrettably, to date, it has not offered advantages in terms of MVA titers when compared to yeast strains and *E. coli*. The highest reported titer to date is 5 g/L, which was achieved using *P. putida* GZT23 cultivated in a 5-L bioreactor [29]. For the methylotrophic bacterium *M. extorquens*, only two reports on MVA production have been documented, with titers only in the range of 2–3 g/L [30,31]. These two types of chassis microorganisms can utilize low-cost carbon sources other than glucose, conferring a distinct cost advantage for industrial biotechnology applications. Nevertheless, achieving higher MVA production levels requires more extensive chassis modifications through multilevel metabolic engineering strategies. Additionally, *Streptomyces lividans* was also tested for MVA biosynthesis. After engineering, the strain only produced 0.66 g/L MVA and required 8 days for fermentation, which is far below industrial requirements. Moreover, genetic manipulation of *Streptomyces* species is more challenging than that of *E. coli*, which results in poor host suitability [32]. These findings indicated that *Streptomyces* species are not ideal hosts for MVA production.

### 2.3 Comparative analysis of different species for MVA synthesis

Among yeast chassis, *S. fibuligera* has mature industrial fermentation experience (maximum titer of 19.4 g/L) but relies on classical screening and mutagenesis rather than genetic engineering, limiting yield scalability [19]. Notably, the strains of this series are not commercially available. *Y. lipolytica* exhibited the highest MVA production capacity, with a reported titer of 101 g/L, although this was supported by only one study [17]. Meanwhile, *S. cerevisiae* exhibited moderate productivity (maximum titer of 13.3 g/L) [15]. It is unsurprising that the MVA production capacity of *S. cerevisiae* is lower than that of *Y. lipolytica*. As a typical oleaginous yeast, *Y. lipolytica* possesses abundant pools of acetyl-CoA, which is a key precursor for MVA biosynthesis, and represents a substantial metabolic advantage. For bacterial chassis, *E. coli* stands out with comprehensive advantages: a high titer (up to 88 g/L), short fermentation cycle (<50 h), and the presence of mature genetic systems [26]. *P. putida* and *M. extorquens* possess unique value in utilizing low-cost non-glucose carbon sources, with *P. putida* additionally exhibiting excellent environmental tolerance. Their major limitation is their low MVA titers (maximum 5 g/L for *P. putida*, 2–3 g/L for *M. extorquens*) [[29], [30], [31]], requiring extensive metabolic engineering for industrial viability. *S. lividans* is unsuitable for MVA production because of its low titer (0.66 g/L), prolonged fermentation (8 d), and poor genetic manipulability.

In summary, *S. fibuligera* remains viable for established industrial processes; *Y. lipolytica* and *E. coli* are the top choices, with *E. coli* offering the advantage of a short fermentation cycle; *P. putida* and *M. extorquens* are strategically promising for sustainable MVA production by utilizing low-value carbon sources, which can reduce reliance on costly glucose feedstocks and enhance economic feasibility (with further metabolic engineering); while *Streptomyces* species are not advisable.

## 3 Research on catalytic enzymes for MVA synthesis

**Fig. 5:**
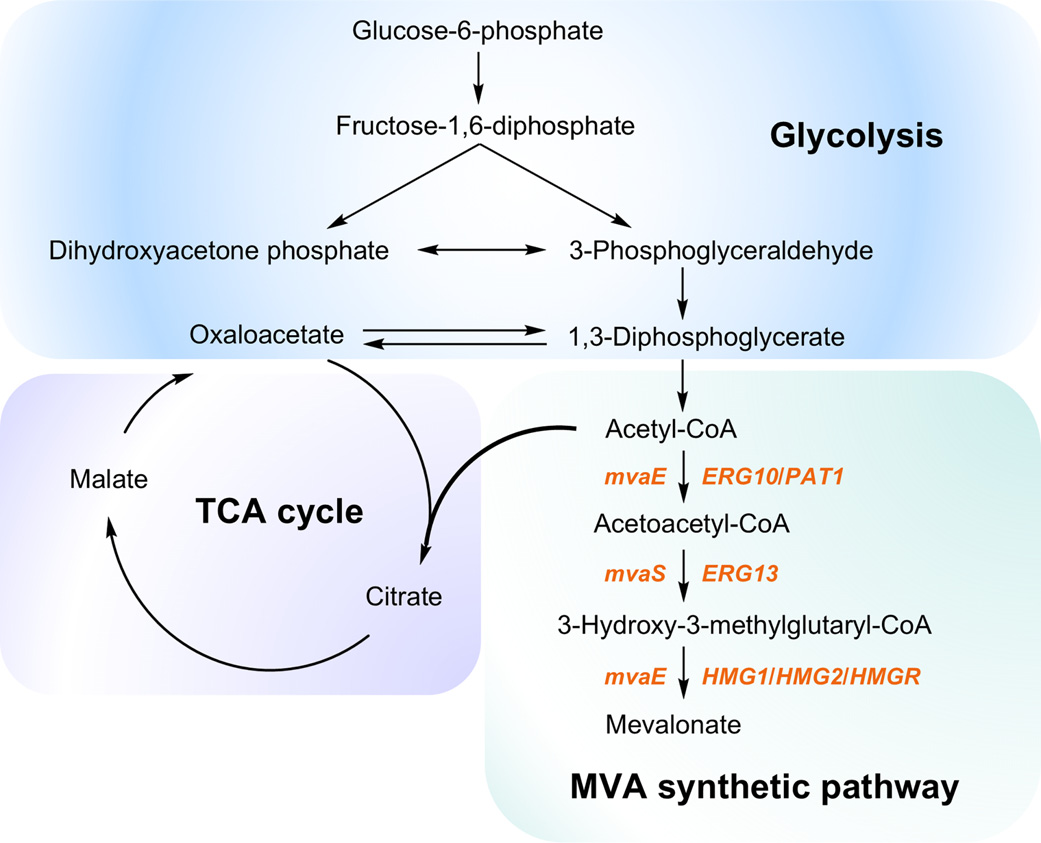
Partial intracellular metabolic pathways associated with MVA biosynthesis. Genes involved in MVA biosynthesis are labeled next to the arrows. The *mvaE* gene encodes a bifunctional enzyme with both AACoAT and HMGR activities; *ERG10*/*PAT1* encode AACoAT; the *mvaS* and *ERG13* genes encode HMGS; *HMG1/HMG2*/*HMGR* encode HMGR.

The MVA biosynthetic pathway encompasses the initial three sequential reactions of the complete intracellular MVA pathway (Fig. 5). First, acetyl-CoA acetyltransferase (AACoAT) catalyzes the condensation of two acetyl-CoA molecules to form acetoacetyl-CoA. Subsequently, 3-hydroxy-3-methylglutaryl-CoA synthase (HMGS) converts acetoacetyl-CoA to 3-hydroxy-3-methylglutaryl-CoA (HMG-CoA), which is further reduced to MVA by HMG-CoA reductase (HMGR) with the consumption of 2 molecules of NAD(P)H + 2H^+^, with cofactor preference varying by HMGR class: class I enzymes (including the human enzyme) exclusively utilize NADPH [33], while class II HMGRs display specificity for NADH [34,35], NADPH [36], or both [[37], [38], [39]]. Among these enzymes, HMGR is widely recognized as the first rate-limiting enzyme in the regulation of the MVA pathway [17,[40], [41], [42], [43]].

The MVA pathway is evolutionarily conserved across diverse taxa, including mammals, fungi, higher plants, and certain archaea [44]. It was first identified independently in yeast and animal systems by Bloch *et al.* and Lynen *et al*. in 1958 [42], and its role in downstream cholesterol biosynthesis was initially discovered in yeast [45]. *S. cerevisiae*, the most representative eukaryotic model [46], has a well-characterized MVA pathway: AACoAT, HMGS, and HMGR are encoded by *ERG10, ERG13*, and *HMG1*/*HMG2*, respectively. Additionally, the MVA pathway is present in several Gram-positive bacteria [44], such as *E. faecalis* [47], *Lactobacillus casei* [48], *Streptococcus pneumoniae* [49], and *Staphylococcus aureus* [50]. Compatibility between the introduced pathway and the chassis host is a critical determinant of the target product titer in heterologous biosynthesis. This compatibility often follows the “phylogenetically close chassis” principle [51,52], although practical efficacy remains the ultimate criterion. As described in Section 2.2, unlike yeast, *E. coli, P. putida*, and *M. extorquens* lack a complete endogenous MVA pathway. They only harbor AACoAT, whereas HMGS and HMGR are absent. Thus, the heterologous expression of genes encoding HMGS and HMGR is essential for MVA production in these prokaryotic hosts.

To break through enzymatic bottlenecks in MVA biosynthesis and enhance the catalytic efficiency of this pathway, two core strategies targeting key MVA synthetic enzymes have been explored: screening of highly active enzymes from diverse species (tailored to specific chassis) and rational enzyme engineering. The succeeding sections contain a detailed summary of the advances made in these two areas.

### 3.1 Screening of chassis-adapted enzymes from diverse species

#### 3.1.1 Enzyme screening for E. coli chassis

Early efforts to reconstitute the MVA pathway in *E. coli* focused on screening enzymes from diverse sources. Yang *et al*. [53] expressed HMGSs and HMGRs from three bacterial species: *S. pneumoniae, S. aureus*, and *E. faecalis*. The *S. pneumoniae* and *S. aureus* pathways co-express the *phaA* gene from *Ralstonia eutropha* (encoding AACoAT), as this heterologous enzyme exhibits higher catalytic activity than the endogenous *E. coli* AACoAT [54]. Notably, *E. faecalis* encodes a bifunctional enzyme MvaE (catalyzing both AACoAT and HMGR activities) and a monofunctional HMGS MvaS, which are encoded by the *mvaE* and *mvaS* genes, respectively [36,55], enabling MVA biosynthesis with only two genes. The *E. faecalis* pathway outperformed the other two sources by a large margin, achieving an MVA titer of 3.93 g/L, compared to only 602 mg/L and 65.7 mg/L for the *S. pneumoniae* and *S. aureus* pathways, respectively. This marked superiority established the *E. faecalis* MVA pathway as the gold standard for subsequent studies [56]. Subsequent studies identified the optimal enzyme combination for *E. coli*. Xiong *et al*. [26] systematically screened MvaS and MvaE from five sources (*E. faecalis, S. aureus, L. casei, Methanococcus maripaludis*, and *Methanococcus voltae*) in *E. coli* BW25113. All the tested heterologous enzymes exhibited activity, except for MvaS from the two *Methanococcus* species, and the combination of *L. casei mvaS* and *mvaE* was the most effective. The strain produced 14.6 g/L MVA from 40 g/L glucose in shake flasks, and this titer was scaled up to 88 g/L in a 1.3-L bioreactor.

Reports on the heterologous expression of yeast-derived MVA biosynthetic pathway genes in *E. coli* with the aim of MVA production remain scarce. Pitera *et al*. [57] overexpressed the *MevT* operon, which comprises *E. coli atoB* and *S. cerevisiae HMGS*/*tHMGR*, in *E. coli*. Subsequent amplification of the *tHMGR* gene enabled the engineered strain to achieve an MVA titer of 4.2 g/L. Furthermore, Yang *et al*. [53] validated the species-specific compatibility of MVA biosynthetic pathways *via* comparative analysis; the *E. coli* strain harboring *E. faecalis mvaS* and *mvaE* accumulated up to 1.31 g/L MVA, which was approximately 50-fold higher than the titer (0.026 g/L) achieved by a strain expressing the *S. cerevisiae*-originated counterpart. These results suggest that the pathway from *E. faecalis* was more effective than that from *S. cerevisiae* in an *E. coli* chassis.

#### 3.1.2 Enzyme selection in other prokaryotic chassis

For *P. putida*, most early studies focused on the *E. faecalis* pathway. In three investigations using *P. putida* KT2440 as the chassis, researchers introduced *E. faecalis mvaS* and *mvaE* [[58], [59], [60]]. In a separate study using a different *P. putida* strain (GZT23) as the host, Zhang *et al*. [29] adopted an alternative gene combination, expressing *E. coli atoB* (AACoAT) together with *L. casei mvaS* and *mvaE*. For the less commonly used prokaryotic chassis *M. extorquens* AM1, Zhu *et al*. [31] compared two pathway modules by evaluating a natural operon (*MVE*) harboring *E. faecalis mvaES* and an artificial operon (*MVH*) encoding HMGS from *Blattella germanica* (*hmgcs1*) and tHMGR (truncated feedback-insensitive form of HMGR) from *Trypanosoma cruzi* (*tchmgr*). At the shake-flask scale, *MVH* yielded 66 mg/L MVA, slightly exceeding that produced by *MVE* (56 mg/L). Co-expression of *R. eutropha phaA* (encoding AACoAT) further increased the titer to 180 mg/L.

#### 3.1.3 Enzyme choice for eukaryotic chassis

Eukaryotic chassis with endogenous MVA pathways often employ two distinct modification strategies: tuning native enzymes or introducing heterologous modules. For *S. cerevisiae*, a model eukaryote with a well-characterized native MVA pathway, researchers have explored both approaches. Rodriguez *et al*. [23] integrated *E. faecalis mvaE* and *mvaS* into *S. cerevisiae* to enhance the flux of cytoplasmic acetyl-CoA toward MVA. In another study, Wegner *et al*. [15] conducted a comparative analysis of this heterologous *E. faecalis mvaE*/*mvaS* module and the yeast endogenous MVA pathway. Although these two systems achieved comparable MVA titers, the heterologous module was less subject to endogenous regulatory constraints. This advantage solidified its status as the preferred foundational biosynthetic unit. However, no studies have reported the molecular modification of the endogenous MVA pathway or the introduction of heterologous modules in *S. fibuligera*. Similarly, in *Y. lipolytica*, researchers have focused on overexpressing native MVA pathway structural genes, rather than adopting heterologous pathways [17].

#### 3.1.4 Comparative analysis of common heterologous MVA pathways

Based on the enzyme screening results across prokaryotic and eukaryotic chassis (Sections 3.1.1 to 3.1.3), four major heterologous MVA pathways have been validated for MVA biosynthesis, with performances differing significantly due to chassis compatibility and catalytic adaptability.

The *E. faecalis*-derived pathway simplifies expression with two genes, adapting to *E. coli, P. putida*, and *S. cerevisiae*, although its 65 g/L titer in *E. coli* is lower than that of the *L. casei*-derived module (88 g/L), and performs poorly in non-model hosts, such as *M. extorquens* AM1 [9,26,31]. The *L. casei* pathway excels in catalytic efficiency, achieving the highest titer in model prokaryotes and stable scale-up, but is limited to Gram-negative bacteria [26,29,61]. The *S. cerevisiae*-derived pathway shows strong homologous compatibility in yeast but poses substantial risks for heterologous expression in prokaryotes. *E. coli* harboring the pathway yielded only 0.026 g/L MVA (50-fold lower than the *E. faecalis* pathway) [53]. The eukaryotic artificial *MVH* pathway (from *B. germanica* and *T. cruzi*) outperforms bacterial pathways in *M. extorquens* (66 mg/L) and enables methanol-based production; however, its productivity remains suboptimal for practical industrial applications [31]. Overall, the pathway selection depends on the chassis type: the *L. casei* pathway is preferred for high-titer prokaryotic production, the *E. faecalis* module for cross-species applications, the *S. cerevisiae* pathway for yeast homologous engineering, and the *MVH* pathway when using the methylotrophic bacterium *M. extorquens*. Optimal biosynthetic performance relies on aligning pathway properties with the metabolic advantages of the host chassis.

### 3.2 Enzyme engineering for the MVA pathway

While screening naturally identified high-activity enzymes, rational engineering further optimizes their catalytic properties to overcome bottlenecks in MVA biosynthesis. However, to date, structure-guided or rational engineering studies targeting the MVA pathway enzymes are relatively limited. For the *mvaE* and *mvaS* genes derived from *E. faecalis*, Steussy *et al*. [62] investigated the spatial structures of their encoded proteins (MvaE and MvaS, respectively). They found that the HMGS encoded by *mvaS* contains a catalytic loop with a nucleophilic cysteine residue, a feature that is both uncommon and relatively unstable. To address this issue, they introduced a point mutation, substituting alanine (A) at position 110 with glycine (G) (designated A110G), which converted the catalytic loop into a more common and stable structure. Enzymatic kinetic assays revealed that the reaction rate of the A110G-mutated HMGS increased approximately 140-fold, providing novel insights for MVA biosynthesis research. Subsequently, Yang *et al*. [53] adopted this mutation strategy by introducing an A110G substitution into *mvaS*, which resulted in a 1.37-fold increase in the MVA titer.

## 4 Metabolic engineering strategies for enhanced MVA production

### 4.1 Transcriptional regulation of MVA structural genes

#### 4.1.1 Yeasts: endogenous transcriptional tuning vs. heterologous module overexpression

In the yeast chassis, the transcriptional regulation of MVA pathway genes relies primarily on two strategies: native promoter replacement and heterologous module integration. In *Y. lipolytica*, the strong constitutive P*_TEFin_* promoter is widely used to drive the expression of endogenous MVA pathway genes (*HMGR, ERG13, ERG10*/*PAT1*) by replacing their native promoters. The overexpression of HMGR (a rate-limiting enzyme) *via* P*_TEFin_* enhanced its transcriptional level by 32.8-fold, leading to a 2,095% increase in MVA titer, whereas ERG13 overexpression improved MVA production by >95%. ERG10/PAT1 overexpression showed no significant titer improvement, likely because acetyl-CoA was not a metabolic bottleneck under the conditions tested [17]. Both heterologous module integration and endogenous pathway tuning have been validated in *S. cerevisiae*. Rodriguez *et al*. [23] expressed *E. faecalis mvaE*/*mvaS via* a galactose-inducible pESC-URA vector (P*_GAL10_* for *mvaE* and P*_GAL1_* for *mvaS*) requiring 2% galactose induction. Based on the pJLA vector series [63], Wegner *et al*. [15] further compared two genome-integrated systems in *S. cerevisiae* CEN.PK2-1C: one harboring *E. faecalis*-derived *mvaE* (driven by P*_GPD1_*) and *mvaS* (driven by P*_TEF1_*), and the other overexpressing endogenous *ERG10* (driven by P*_GPD1_*), *ERG13* (driven by P*_TEF1_*), and *HMG1* (driven by P*_TEF1_*). Both systems achieved comparable MVA yields, and constitutive overexpression of the pathway enabled the production of significant levels of MVA, whereas wild-type or empty vector-containing control strains produced little or no MVA [15].

#### 4.1.2 E. coli: promoter types, vector systems and chromosomal integration

Most structural genes for MVA biosynthesis in *E. coli* are overexpressed *via* plasmids, such as pACYCduet-1 [64,65] and pTrc99A [56], whose transcription is typically driven by vector-borne inducible promoters (e.g., T7, *trc* promoter (P*_trc_*)). For example, Dong *et al*. [65], Yang *et al*. [53], and Xu *et al*. [66] used the T7 promoter in pACYCduet-1 to drive *mvaE*/*mvaS* expression, which required IPTG induction. Satowa *et al*. [67] employed IPTG-inducible P*_trc_* in pTrcHisB for the expression of key MVA pathway genes (*atoB, mvaS*, and *mvaA*), while Kamata *et al*. [68] adopted the *trp* promoter (P*_trp_*) in pUA66-P*trp*-*mvaES* with 10 μM 3β-indoleacrylic acid to enhance promoter activity [69]. In *E. coli* XL1-Blue, the expression plasmid for the MVA pathway genes was constructed based on pTrS30, with *mvaS* and *mvaE* co-controlled by a single P*_trp_* promoter, whose activity was regulated by the tryptophan concentration in the medium [61]. In *E. coli* BW25113, core MVA pathway genes (*atoB, mvaS*, and *mvaE*) were all located downstream of the IPTG-inducible *P_L_*lacO1, and their transcription was exclusively driven by this promoter [26].

As is widely recognized in heterologous pathway engineering, merely stacking genes together is not sufficient; rather, the precise matching of enzyme activities is required. Even if the activity of a single enzyme is extremely high, insufficient consumption of its product by downstream enzymes can lead to the accumulation of toxic intermediates, ultimately reducing the target product yield. Many studies have focused solely on “enhancing pathway gene expression,” while neglecting “the metabolic flux balance”. For MVA biosynthesis in *E. coli* chassis, the toxicity of intermediates is an underappreciated risk in heterologous expression. Pitera *et al*. [57] demonstrated that excessive expression of the *MevT* operon, which encodes enzymes responsible for MVA biosynthesis, led to the toxic accumulation of HMG-CoA in an *E. coli* chassis. This was resolved by tHMGR overexpression, which enhanced HMG-CoA conversion, doubled MVA titer, and restored growth. In another study, using *E. coli* BL21 as the chassis, *mvaE* and *mvaS* were initially placed downstream of independent T7 promoters. To address “the precursor imbalance” (a 1:1 ratio of acetyl-CoA to acetoacetyl-CoA is required for condensation into HMG-CoA), Jeung *et al*. [64] optimized the promoter system by replacing the T7 promoter driving *mvaE* with the anhydrotetracycline (aTc)-inducible Tet promoter, while retaining the T7 promoter controlling *mvaS*. This “dual-promoter and dual-inducer” design allowed the precise control of *mvaE* and *mvaS* expression by adjusting the concentrations of aTc (regulating *mvaE*) and IPTG (regulating *mvaS*), thereby balancing their expression. Finally, MVA titer was increased from 0.19 g/L to 0.7 g/L.

Some studies have noted that current strategies relying on inducible promoters and the overexpression of MVA pathway genes *via* episomal plasmids present several notable limitations. These include high costs due to the requirement for expensive inducers, such as IPTG and arabinose, which render large-scale production economically unfeasible. Additionally, they impose a heavy metabolic burden, as inducers may interfere with host cell metabolism and even cause heterogeneous gene expression at the single-cell level, whereas plasmid replication and the expression of plasmid-borne genes consume cellular carbon and energy, diverting resources away from MVA biosynthesis. Moreover, these strategies are highly dependent on the induction conditions, necessitating strict control over the inducer concentration and induction timing, which increases operational complexity. Finally, genetic instability arises from uneven plasmid segregation during bacterial cell division, leading to the accumulation of plasmid-free cells and ultimately reducing product yield [24]. To address these issues, constitutive promoters coupled with either chromosomal integration or plasmid-based maintenance of MVA pathway genes have been proposed. Such strategies eliminate the need for inducers and enable continuous gene expression throughout cell growth, with chromosomal integration further enhancing genetic stability. Guo *et al*. [70] evaluated 10 glycolytic promoters in *E. coli* BL21(DE3) and found that 7 high-strength constitutive promoters (e.g., P*_gap_*, P*_glk_*) achieved much higher MVA titers (up to 4.28 g/L for P*_gap_*) than the *lac* promoter control, without the need for inducers. Wang *et al*. [71] replaced the IPTG-inducible *tac* promoter with Anderson promoters of varying strengths to regulate the expression levels of MVA pathway genes. This adjustment matched the supply of acetyl-CoA and NADPH from the EP-bifido pathway, avoiding the metabolic burden or insufficient flux caused by excessively high or low gene expression. Another study [61] integrated the core MVA pathway genes (*atoB, mvaS* and *mvaE*) into the *E. coli* BW25113 chromosome (driven by the constitutive promoter M1-93 [72]), replacing *adhE* and *ldhA* to reduce byproduct formation. The final strain yielded 30 g/L MVA in shake flasks, which was significantly higher than the 14.6 g/L from episomal plasmids [26].

#### 4.1.3 M. extorquens AM1: methanol-dependent transcription

For *M. extorquens* AM1, core genes of the MVA pathway were all expressed *via* the pCM110 vector [31], which contains the default expression element, the P*_mxaF_* promoter [73]. Derived from *mxaF* (a key gene in methanol metabolism that encodes the large subunit of methanol dehydrogenase) [74], the P*_mxaF_* promoter is methanol-dependent and can stably drive gene expression when methanol serves as a carbon source. Furthermore, to achieve stable expression, the entire MVA pathway including all core and auxiliary genes overexpressed using the pCM110 backbone, was integrated into the *attTn7* site of the chassis genome *via* homologous recombination [30].

#### 4.1.4 P. putida: inducible promoters and their vector-based application in core MVA genes

In two studies by Yang *et al*. [59,75], the *E. coli*-*Pseudomonas* shuttle expression vector pUCP19 was explicitly modified by inserting the *lacI*+P*_trc_* fragment derived from the pTrc99A vector. The P*_trc_* promoter drove the expression of core MVA biosynthesis genes (*atoB, mvaS*, and *mvaE*). All cultivation experiments in these studies required the addition of 0.1 mM IPTG at the initiation of cultivation for P*_trc_* activation. Similarly, Kim *et al*. [58] used the pSEVA231 vector [76] in the *P. putida* KT2440 chassis, in which *mvaE* and *mvaS* were still driven by the P*_trc_* promoter. In summary, the transcriptional regulation of MVA structural genes is highly chassis-specific: yeasts mainly adopt native promoter replacement and heterologous module integration; *E. coli* focuses on promoter/vector optimization and chromosomal integration to balance flux and reduce metabolic burden, whereas *M. extorquens* AM1 and *P. putida* rely on methanol-dependent and inducible promoters, respectively, to adapt to their unique metabolic characteristics. The core goal of these strategies is to achieve efficient, stable, and metabolically compatible expression of the MVA pathway genes.

### 4.2 Translational optimization for metabolic flux balance

Translational optimization primarily relies on ribosome-binding site (RBS) engineering to modulate enzyme expression strength, thereby balancing the MVA pathway flux and avoiding issues such as precursor shortages or intermediate accumulation caused by unbalanced enzyme activities. This strategy has been validated in prokaryotic microbial chassis, either as a standalone approach or in combination with transcriptional regulation. In *M. extorquens* AM1, the exogenous expression of the *phaA* gene (encoding AACoAT) led to suboptimal MVA production when its enzymatic activity was either too weak or too strong. Insufficient expression limits the supply of precursor molecules, whereas excessive expression competes for metabolic resources and impairs cell growth. Replacement of the native RBS of *phaA* with variants of different strengths resolved this imbalance; a moderate-strength RBS (AM1-ARBSU1) achieved optimal *phaA* expression and an MVA titer of 215 mg/L, which was superior to weak (170 mg/L) or overly strong (no further improvement) RBS variants [31]. This principle was further extended to RBS engineering of *HMGR* in *M. extorquens* AM1, achieving additional optimization of the MVA biosynthesis pathway [30]. For *P. putida*, a combined strategy of “promoter screening and RBS engineering” was adopted to balance the metabolic flux. After identifying the high-activity constitutive promoter J23111 that drives the core MVA pathway operon (*atoB, mvaS, mvaE*) at the transcriptional level, RBS variants of varying strengths (high: BBa_B0030; medium: BBa_J61106; low: BBa_J61109) were paired with AtoB, MvaS, and MvaE to optimize the translational levels. The optimal combinations (AtoB with medium-activity RBS, MvaS with low-activity RBS, and MvaE with high-activity RBS) prevented HMG-CoA accumulation and achieved a shake-flask MVA titer of 470 mg/L [29]. Translational optimization *via* RBS engineering is an effective strategy for balancing MVA pathway flux. By adjusting the expression strength of key enzymes, it avoids problems such as precursor shortage or intermediate accumulation caused by unbalanced enzyme activities, thereby improving the MVA production efficiency in the microbial chassis.

### 4.3 Precision activation of the MVA pathway *via* CRISPR-Cas transcriptional activation (CRISPRa)

Beyond traditional promoter/RBS engineering, CRISPRa has emerged as a novel tool for the targeted overexpression of MVA structural genes in *P. putida* KT2440, addressing the leaky expression issue of inducible promoters. J3-BBa_J23117, a weak promoter in the Anderson promoter collection, exhibits low basal expression and is readily activated by CRISPRa in *P. putida* KT2440. In a study by Kiattisewee *et al.*, it served as the core regulatory element for MVA pathway genes (*mvaE* and *mvaS*) [60]. Specifically, CRISPRa targeted a specific site upstream of this promoter *via* J306 scaffold RNA, recruited the dCas9/MCP-SoxS activation complex, and initiated the transcription of *mvaE* and *mvaS*. The control system was driven by the IPTG-inducible *lacI*-P*_trc_* promoter to drive *mvaE* and *mvaS* expression, consistent with the target genes of the CRISPRa-mediated system. The results showed that the *lacI*-P*_trc_* system exhibited significant leaky expression (MVA titer reached 214 ± 57 mg/L in the absence of IPTG) and large fluctuations in titer post-IPTG induction (66–459 mg/L). In contrast, the CRISPRa system utilizing the J3-BBa_J23117 promoter achieved leak-free expression and stable activation of MVA pathway genes with stable MVA production (402 ± 21 mg/L). Collectively, CRISPRa technology enables precise and stable activation of the MVA pathway by overcoming the leaky expression defects of traditional inducible promoters. This provides a reliable and controllable regulatory tool for the metabolic engineering of MVA production, especially in the chassis, where precise gene expression is critical.

### 4.4 Spatial organization of MVA pathway enzymes

Natural biosynthetic pathways often rely on the spatial clustering of enzymes to enhance intermediate channeling and reduce toxic accumulation [77]. This principle has been mimicked *via* synthetic scaffolds to optimize MVA production. Two major scaffold types, protein- and RNA-based, have been validated with remarkable efficiencies, as detailed below. Fink *et al*. designed orthogonal coiled-coil heterodimers as modular protein scaffolds to cluster MVA enzymes. Three core MVA enzymes (ERG10, HMGS, and tHMGR) were fused with a P3:GCN:P4 coiled-coil scaffold in *S. cerevisiae*. This spatial organization reduced HMG-CoA diffusion and boosted the MVA titer by 8.8-fold compared to non-scaffolded strains [16]. RNA scaffolds offer a flexible alternative to protein scaffolds, leveraging specific RNA-protein interactions for enzyme co-localization. Dong *et al*. developed a 0D PP7 RNA scaffold system consisting of PP7 RNA aptamers and their cognate RNA-binding domains (ΔPP7 RBDs). When applied to the MVA pathway in *E. coli* BL21(DE3) cells, fusing ΔPP7 RBDs to MvaE/MvaS and co-expressing the PP7 RNA scaffold resulted in an 84% increase in the MVA titer. This improvement was attributed to reduced intermediate loss and balanced metabolic flux, highlighting RNA scaffolds as a low-burden option for prokaryotic MVA engineering [65]. Collectively, the spatial organization of MVA pathway enzymes *via* protein or RNA scaffolds effectively enhances intermediate channeling, reduces the accumulation of toxins, and balances metabolic flux. Protein scaffolds have shown remarkable efficacy in yeast, whereas RNA scaffolds provide a low-metabolic-burden option for prokaryotes, both of which represent valuable strategies for improving MVA production.

### 4.5 Enhancing acetyl-CoA precursor supply and NADPH availability

#### 4.5.1 Enhancing acetyl-CoA precursor supply in eukaryotic yeast chassis

*S. cerevisiae* is a commonly used host for metabolic engineering; however, it has the critical limitation of overflow metabolism. Under high-glucose fermentation conditions (typical for industrial processes), over 60% of glucose is converted to ethanol and glycerol (innate metabolic byproducts of yeast that are irrelevant to MVA production). Additionally, *S. cerevisiae* exhibits low acetyl-CoA synthesis efficiency: it relies on the “PDH bypass” to produce cytosolic acetyl-CoA, but the key enzyme acetyl-CoA synthase (ACS) is subject to product feedback inhibition, preventing sustained and efficient acetyl-CoA generation. These issues ultimately lead to insufficient levels of cytosolic acetyl-CoA for MVA biosynthesis. To address this limitation, Rodriguez *et al*. [23] introduced the ATP-citrate lyase (ACL) pathway from oleaginous organisms into *S. cerevisiae* and optimized the metabolic flux *via* metabolic engineering. They tested ACLs from five sources and found that the ACL from *Aspergillus nidulans* exhibited the highest activity. The authors further deleted the mitochondrial *IDH1* gene (encoding NAD^+^-dependent isocitrate dehydrogenase), which blocked isocitrate metabolism in the tricarboxylic acid (TCA) cycle, leading to mitochondrial citrate accumulation, which was then transported to the cytosol at higher rates *via* the citrate transporter (CTP1) to serve as a substrate for ACL. Wegner *et al*. [15] reported that blocking overflow metabolism by deleting *ADH1* (encoding alcohol dehydrogenase) and *GPD1* (encoding glycerol-3-phosphate dehydrogenase) reduced carbon source shunting, leading to a 10-fold increase in the MVA titer. Expressing the ACS mutant *Se-acs*^L641P^ from *Salmonella enterica*, which is insensitive to acetyl-CoA feedback inhibition, and supplementing pantothenate after the overexpression of *CAB1* (encoding pantothenate kinase, the rate-limiting enzyme for CoA biosynthesis) further enhances the intracellular CoA pool content and strengthens acetyl-CoA synthesis efficiency. Similarly, to enhance the biosynthesis of ursolic acid, a downstream product of the MVA pathway, Zhu *et al*. [78] heterologously expressed *Bbfpk* from *Bifidobacterium bifidum* and *Ckpta* from *Clostridium kluyveri*, enabling the direct conversion of glycolytic intermediates to acetyl-CoA and bypassing energy-consuming steps. In addition, they overexpressed *ALD6* (encoding acetaldehyde dehydrogenase) and mutant *Se-acs*^L641P^, which facilitates the efficient conversion of acetate to acetyl-CoA.

Unlike *S. cerevisiae, Y. lipolytica* inherently maintains an abundance of acetyl-CoA pools. As an oleaginous yeast, it converts up to 50% of its dry weight into lipids (with acetyl-CoA serving as a common precursor for both lipids and MVA), making it naturally suitable for producing acetyl-CoA-derived products. However, *Y. lipolytica* exhibits “citrate overflow” under high-yield conditions (e.g., fed-batch fermentation), where large amounts of citrate are secreted into the medium (up to 57.8 g/L). This carbon source waste prevents flux redirection toward MVA. The corresponding optimization strategies focus on mitigating citrate overflow and redirecting the carbon flux toward acetyl-CoA. Specifically, endogenous ACL (ACL1/2) and heterologous high-activity ACL from *A. nidulans* (AnACLa/b) were overexpressed to enhance citrate-to-acetyl-CoA conversion. Additionally, the mitochondrial citrate transporter YHM2 was overexpressed to increase cytosolic citrate flux. Furthermore, the mitochondrial isocitrate dehydrogenase genes (*IDH1*/*IDH2*) were deleted to block citrate catabolism in the TCA cycle, thereby promoting mitochondrial citrate accumulation and export. Finally, *CEX1* (encoding a citrate exporter) was deleted to completely abolish citrate secretion [17]. Following these systematic modifications to the acetyl-CoA precursor supply pathway, the fermentation results showed that the citrate concentration in the medium was only 3.4 g/L, while the MVA titer increased to 101 g/L with a yield of 0.255 g/g glucose, equivalent to 46.8% of the theoretical maximum yield.

#### 4.5.2 Enhancing acetyl-CoA precursor supply in the prokaryotic E. coli chassis

Wang *et al*. [61] noted that the native metabolism of *E. coli* leads to carbon source wastage, which has three key limitations. First, glucose metabolism produces byproducts such as ethanol and lactate, diverting carbon flux away from the target biosynthesis. Second, glycolysis (the first step in acetyl-CoA generation) exhibits a low flux, resulting in an insufficient precursor supply. Third, acetyl-CoA is consumed by the TCA cycle for energy production, rather than being efficiently directed toward MVA synthesis. To address these issues, the MVA pathway gene cluster was integrated into the *E. coli* chromosome to replace *adhE* (encoding alcohol dehydrogenase) and *ldhA* (encoding lactate dehydrogenase). This modification not only increased the copy number of exogenous MVA genes but also reduced the production of ethanol and lactate. Glycolysis serves as the primary source of acetyl-CoA. Bacterial ATP synthase (with the *atpFH* gene encoding its subunits) inhibits glycolysis: accumulated ATP acts as a negative regulator of glycolytic enzyme activity. Thus, the authors deleted the *atpFH* gene, which doubled glycolytic flux and increased MVA productivity from 0.43 g/L/h to 0.92 g/L/h. Furthermore, the *sucA* gene (encoding 2-oxoglutarate decarboxylase, a key enzyme in the TCA cycle) was deleted. This deletion blocks the TCA cycle, preventing acetyl-CoA from being used for energy production and redirecting it exclusively toward MVA synthesis. Notably, 5 mM succinate was added to the culture to mitigate the growth defects caused by TCA cycle disruption. In another study, Jeung *et al*. recognized that the complete deletion of *gltA* (encoding citrate synthase, a critical TCA cycle enzyme) caused growth arrest in *E. coli*. Instead, they used the McbR regulatory system to replace the native promoter of *gltA* with a promoter recognized by McbR (a repressor protein). By expressing varying levels of McbR, the authors achieved moderate repression of *gltA*, a strategy that maximizes the MVA titer while avoiding severe growth impairment [64].

#### 4.5.3 Synergistically enhancing acetyl-CoA precursor supply and NADPH availability in E. coli chassis

Intracellular NADPH availability is a critical cofactor for efficient MVA production. One strategy to boost NADPH supply involves replacing NAD^+^-dependent enzymes with their NADP^+^-dependent counterparts, thereby redirecting metabolic flux toward the NADPH-generating pentose phosphate (PPP) pathway [79]. Additionally, overexpression of the transhydrogenase PntAB has been shown to increase NADPH regeneration, which in turn improves the production of metabolites such as 3-hydroxypropionic acid [80], shikimic acid [81], and agmatine [82]. For MVA biosynthesis in *E. coli*, strategies to enhance NADPH availability are typically paired with acetyl-CoA boosting measures, focusing on resolving the imbalance between these two factors, which is a critical bottleneck for the pathway. In native metabolism, the conversion of glucose to acetyl-CoA requires pyruvate decarboxylation (converting pyruvate to acetyl-CoA and CO_2_). One molecule of glucose yields a maximum of two molecules of acetyl-CoA, accompanied by the release of two molecules of CO_2_, resulting in a theoretical maximum carbon yield of only ∼66%: this not only wastes carbon sources, but also increases greenhouse gas emissions. Furthermore, MVA biosynthesis requires NADPH as a reducing equivalent; however, the Embden–Meyerhof–Parnas (EMP) pathway (the main glycolytic pathway in *E. coli*) produces little NADPH. The supply of NADPH is primarily dependent on the PPP pathway; when the carbon flux is predominantly directed through the EMP pathway, NADPH supply becomes a bottleneck. Kamata *et al*. addressed this issue by precisely regulating the flux ratio between the EMP pathway (which is efficient for acetyl-CoA production) and the oxidative pentose phosphate (oxPP) pathway (which is efficient for NADPH generation). They identified an optimal balance between these two pathways, leading to a 25% increase in MVA titer during the stationary phase [68]. To overcome the limitations of native metabolism (low acetyl-CoA yield, high CO_2_ emissions, and insufficient NADPH), Wang *et al*. designed and constructed a novel metabolic pathway in *E. coli*, the EP-bifido pathway. This pathway integrates three modules: the EMP pathway, PPP, and a bifid shunt (derived from *Bifidobacterium adolescentis*). Specifically, the EMP pathway provides basal carbon flux, the PPP generates NADPH, and the bifid shunt ensures carbon conservation. This synergistic closed-loop system resolves the limitations of native metabolism and significantly enhances MVA production [83]. In a follow-up study, Li *et al*. [71] further optimized the EP-bifido pathway by addressing two key bottlenecks: insufficient PPP flux (leading to NADPH shortage) and growth defects caused by the deletion of *pfkA* (encoding 6-phosphofructokinase). They employed two precise regulatory strategies: strengthening the PPP metabolic flux by replacing the native promoter of the *zwf* gene (encoding glucose-6-phosphate dehydrogenase, a key PPP enzyme) with a strong promoter and dynamically and moderately repressing the EMP pathway using a CRISPR interference (CRISPRi) system. These modifications increased the shake-flask MVA yield to 68.7%. Satowa *et al*. investigated whether NADPH acts as a rate-limiting factor for MVA production by deleting genes at the branch point of the glycolytic/PPP pathways to enhance PPP flux (and thus NADPH generation). Their results confirmed that NADPH is not the rate-limiting factor. The authors then shifted their focus to blocking acetyl-CoA diversion by deleting genes involved in the TCA cycle or related pathways to reduce acetyl-CoA consumption. The found that the deletion of *gltA* (encoding citrate synthase) yielded the most significant improvement, directly blocking the entry of acetyl-CoA into the TCA cycle and redirecting it exclusively toward MVA synthesis, resulting in an MVA titer of 7.19 g/L. The deletion of *ppc* (encoding phosphoenolpyruvate carboxylase) had a less pronounced effect; it reduced oxaloacetate production, indirectly weakened the TCA cycle, and promoted acetyl-CoA accumulation. In contrast, the deletion of *aceBA* (encoding enzymes of the glyoxylate shunt) had no effect, as the glyoxylate shunt consumes minimal acetyl-CoA, and its blockage does not provide a notable benefit [67]. Similarly, in *E. coli* DH5α, researchers tested whether NADPH alone was a bottleneck by overexpressing six genes predicted to enhance NADPH availability. Their results indicate that NADPH was not the sole bottleneck. To enhance the supply of acetyl-CoA, the authors introduced an artificial phosphoketolase-phosphotransacetylase (xPK-PTA) bypass, which circumvents the decarboxylation steps to ensure carbon conservation. Introduction of the xPK-PTA bypass altered metabolic flux; more carbon was directed through the hexose monophosphate (HMP) pathway, increasing NADPH demand (thus consuming previously excess NADPH). When the authors again attempted to overexpress the NADPH-related genes, the results were markedly different. Overexpression of *gnd* (encoding 6-phosphogluconate dehydrogenase, a key PPP enzyme) increases the NADPH/NADP^+^ ratio by 1.7-fold, leading to an MVA titer of 6.37 g/L [56]. Overall, enhancing acetyl-CoA and NADPH availability is a core strategy for improving MVA production. Eukaryotic yeasts mainly optimize acetyl-CoA supply by regulating citrate transport and blocking overflow metabolism, whereas *E. coli* adopts gene deletion, artificial pathway construction, and flux ratio regulation to synergistically improve precursor supply and cofactor balance. These strategies effectively address the metabolic bottlenecks caused by insufficient acetyl-CoA and NADPH, thereby significantly promoting MVA biosynthesis.

### 4.6 Attenuating the downstream branch of the MVA biosynthesis pathway

Attenuation of the downstream branch of the MVA biosynthesis pathway primarily occurs in the yeast chassis. Unlike several commonly used prokaryotic chassis, which lack downstream MVA pathways and thus do not consume MVA as a carbon source, the eukaryotic yeast chassis further metabolize MVA, which is converted to mevalonate 5-phosphate by MVA kinase (encoded by *ERG12*) and ultimately flows toward ergosterol (a membrane component essential for yeast viability but unnecessary for industrial production) *via* squalene synthase (encoded by *ERG9*). Therefore, enabling the accumulation of MVA is critical. In *S. cerevisiae*, Rodriguez *et al*. implemented a promoter replacement strategy, substituting the native promoter of *ERG12* with a copper-repressible *CTR3* promoter (P*_CTR3_*). The P*_CTR3_* promoter is repressed in the presence of copper ions, resulting in *ERG12* silencing and activated in their absence restoring *ERG12* expression. This allowed the controlled attenuation of ERG12 activity, retaining minimal activity to meet the sterol requirements of yeast without impairing growth, while reducing MVA flux into downstream pathways [23]. Jakounas *et al*. developed an efficient, off-target-free CRISPR/Cas9 tool that enabled the simultaneous editing of up to five different genomic loci in *S. cerevisiae*, which was used to screen yeast strains with high MVA production. The authors targeted five key nodes that influenced the MVA flux. In addition to two genes of unknown function (*ypl062w* and *yjl064w*) and the transcriptional repressor *rox1* (detailed in Section 4.8), they included *bts1* (encoding geranylgeranyl pyrophosphate synthase, a branch point diverting MVA toward isoprenoids), whose deletion promoted MVA accumulation and targeted the promoter of *ERG9*, where the truncation of the upstream activation sequence region reduced ERG9 expression, thereby decreasing MVA flux toward ergosterol [22]. Similarly, Wegner *et al*. used CRISPR to replace the native *ERG9* promoter with the methionine-repressible P*_MET3_* promoter in their study [15]. In strains with enhanced acetyl-CoA supply, *ERG9* repression increased the MVA titer from 1.62 g/L to 3.81 g/L, representing the most impactful single modification in that study. A similar strategy was used for *Y. lipolytica*. From the *Y. lipolytica* transcriptome, the authors selected promoters with <50% activity of the native *ERG12* promoter, no stress responsiveness, and short length (<1000 bp). The P3 promoter was selected for this study. Replacing the native *ERG12* promoter with P3 prevented MVA catabolism: *ERG12* expression was significantly reduced, MVA was no longer rapidly metabolized, and the MVA titer increased from 0.02 g/L in the parental strain ZG03 to 0.13 g/L (a 5.5-fold improvement) without impairing late-stage yeast growth [17]. In brief, attenuating the downstream branch of the MVA pathway is a key strategy for yeast chassis, as it reduces the diversion of MVA toward non-target metabolites (e.g., ergosterol). Through promoter replacement, CRISPR-mediated gene expression inhibition, or deletion of branch point genes, MVA accumulation is effectively promoted while maintaining the basic growth needs of yeast, which is crucial for achieving high MVA yields in yeast.

### 4.7 CRISPR-mediated regulation of transcriptional repressors for MVA production

In *S. cerevisiae*, the CRISPR/Cas9 system has been used to screen for yeast strains with high MVA production. Among the five selected editing targets, ROX1 (a transcriptional repressor) [84] represses key genes in the MVA pathway, while the deletion of *rox1* activates the MVA pathway [22]. In *P. putida*, the *glpR* gene (encoding a transcriptional repressor) represses key glycerol metabolism genes (*glpFKRD*). This repression leads to a prolonged 19-h lag phase when glycerol is used as the carbon source, accompanied by slow cell growth and carbon source waste. To address this issue, Kim *et al*. developed a simple, leak-free, and efficient CRISPRi system for *P. putida* KT2440. Targeting *glpR via* CRISPRi yielded multiple improvements: specifically, the lag phase was shortened from 19 h to 9 h, enabling cells to rapidly enter the exponential growth phase; furthermore, cell growth was accelerated, with the final OD₆₀₀ increasing from 1.14 to 2.22; additionally, glycerol consumption was enhanced, rising from 0.96 g/L to 3.14 g/L. By relieving this repressive “brake” on glycerol metabolism, CRISPRi completely resolved the bottleneck of glycerol utilization in *P. putida*. Notably, this CRISPRi-based strategy offers greater flexibility than the traditional *glpR* knockout, as the repression strength can be regulated *via* rhamnose, which significantly improves the MVA titer. Overall, the CRISPR-mediated regulation of transcriptional repressors provides an efficient means to enhance MVA production. Moreover, the deletion of repressors of the MVA pathway (e.g., ROX1 in *S. cerevisiae*) directly activates the pathway, while inhibiting repressors of carbon source metabolism (e.g., *glpR* in *P. putida*) optimizes nutrient utilization. This strategy offers high specificity and flexibility, and effectively overcomes metabolic bottlenecks in diverse chassis.

## 5 Carbon source selection for prokaryotic chassis (*E. coli* and *P. putida*)

For MVA production *via E. coli* fermentation, glucose is the most commonly used carbon source for MVA production; however, other carbon sources have also been investigated. Wang *et al*. [56] utilized low-cost glycerol as the carbon source for MVA synthesis in the *E. coli* DH5α chassis. However, two core bottlenecks previously limited MVA production from glycerol in *E. coli*: carbon loss during acetyl-CoA synthesis and an imbalance in NADPH supply and demand. Specifically, MVA biosynthesis requires two NADPH per MVA molecule, with NADPH primarily supplied by the PPP pathway. An insufficient PPP flux during glycerol metabolism often leads to a shortage of NADPH. To address these issues, Wang *et al*. introduced an xPK-PTA carbon-conserving bypass to resolve acetyl-CoA deficiency and co-overexpressed *gnd* to supplement NADPH. This combination ultimately achieved an MVA titer of 7.21 g/L (see Section 4.6.3). Satowa *et al*. expanded the use of cellobiose as a direct carbon source for MVA production. Lignocellulose is an inexpensive and renewable resource, but its degradation product cellobiose (a glucose dimer), cannot be directly utilized by *E. coli* and requires hydrolysis by β-glucosidase (BGL) to glucose. The authors displayed BGL on the surface of *E. coli* cells, enabling the direct utilization of cellobiose for MVA synthesis [67]. Low-cost acetate is another potential carbon source, as it can be directly converted to acetyl-CoA precursor *via* the ACS and phosphotransacetylase/acetate kinase (Pta-AckA) pathways [66,85]. However, *E. coli* exhibits low natural efficiency of acetate metabolism, and acetate is toxic to these cells. Xu *et al*. addressed acetate toxicity by optimizing the fermentation strategy. They employed two-stage fermentation with additional glucose as the co-carbon source (see Section 6 for details) [66]. In contrast, Jeung *et al*. used a repeated fed-batch strategy to produce MVA using acetate as the sole carbon source [64].

*P. putida* KT2440 can grow on various carbon sources. In addition to conventional glucose, ethanol [75], glycerol [58], and 2,3-butanediol (2,3-BDO) [59] have been explored as carbon sources for MVA synthesis. Glycerol, an inexpensive byproduct of the biodiesel industry, is an ideal low-carbon feedstock. However, the *glpR* gene in *P. putida* (encoding a transcriptional repressor) inhibits key glycerol metabolism genes (*glpFKRD*), leading to a prolonged lag phase (19 h), slow cell growth, and carbon source waste when glycerol is used. To resolve this issue, the authors developed a single-plasmid CRISPRi system to optimize glycerol metabolism by regulating *glpR* expression, ultimately enhancing MVA production [58] (see Section 4.8 for details). Yang *et al*. were the first to use *P. putida* as a chassis for efficient MVA production from ethanol. Ethanol offers the advantage of direct conversion to acetyl-CoA without carbon loss. Further metabolic engineering modifications resulted in a shake-flask MVA yield of 0.41 g/g ethanol (the highest reported yield for non-carbohydrate carbon sources at the time) and a batch fermentation titer of 4.60 g/L [75]. Subsequently, Yang *et al*. achieved the direct conversion of 2,3-BDO to MVA. *P. putida* naturally metabolizes 2,3-BDO and leverages the advantage of 2,3-BDO’s carbon-loss-free conversion to acetyl-CoA; additional metabolic engineering yielded a shake-flask MVA titer of 2.21 g/L (with a yield of 0.295 g/g 2,3-BDO, equivalent to 27% of the theoretical maximum). Notably, MVA production using 2,3-BDO as the carbon source was 6.61-fold and 8.44-fold higher than using glucose and glycerol, respectively [59]. In summary, the selection of microbial carbon sources for MVA production typically requires systematic coordination with chassis metabolic engineering to ensure efficient carbon utilization.

## 6 Fermentation regulation for increased MVA production

Regulation of fermentation for increased MVA synthesis mainly includes process control and regulation of factors influencing fermentation, which, in turn, can regulate cellular metabolism to promote MVA production. In a study in which Yang *et al*. engineered *P. putida* KT2440 to convert 2,3-BDO to MVA, the aeration rate (shaking flask rotation speed, rpm) affected dissolved oxygen (DO) levels, which in turn determined the flux of acetyl-CoA. Thus, optimizing the aeration rate is critical for balancing cell growth and product synthesis. At high aeration rates (300 rpm), sufficient DO preferentially directs acetyl-CoA toward the TCA cycle to support cell growth, resulting in an extremely low MVA titer (≈ 0 g/L). At low aeration rates (150 rpm), limited DO reduced TCA cycle activity, diverting more acetyl-CoA toward MVA biosynthesis. At excessively low aeration rates (≤100 rpm), insufficient DO inhibited cell growth and prevented the complete consumption of 2,3-BDO, leading to decreased MVA production. The optimal rotation speed was found to be 150 rpm, resulting in an MVA titer of 2.21 g/L and moderate cell growth (OD_600_ ≈ 3.5). This balances biomass accumulation with minimal acetyl-CoA waste [59]. In another study, Xu *et al*. [66] used low-cost acetate as a carbon source for efficient MVA production in recombinant *E. coli* cells. They first tested single-carbon-source fed-batch fermentation with acetate, continuously supplying acetate in a 5-L bioreactor. As expected, the toxic effects of acetate inhibited bacterial growth. To address this, they developed a two-stage fed-batch fermentation strategy. During phase 1 (the growth phase), glucose was supplied to facilitate robust bacterial growth, achieving a biomass of 8.6 g/L. In phase 2 (the production phase), acetate and the inducer IPTG were added after glucose depletion to halt further biomass accumulation and redirect carbon flux from primary metabolism to secondary MVA synthesis. This strategy increased the MVA titer from 1.06 g/L (a single acetate carbon source) to 7.85 g/L. Given that oxygen acts as a “metabolic energy switch” for bacteria, three oxygen conditions were further tested under the two-stage fed-batch mode: aerobic conditions (continuous aeration, DO = 20%) yielded the highest MVA titer (7.85 g/L), as active bacterial metabolism supported increased MVA synthesis. Anaerobic conditions (no aeration, nitrogen sparging) achieved the highest substrate utilization efficiency (0.37 g MVA/g acetate), since limited oxygen prevented acetate waste in biomass accumulation or energy production and diverted more acetate directly to MVA. Microaerobic conditions (limited aeration, 1.0 vvm) resulted in an intermediate MVA titer (4.97 g/L). These results confirm that oxygen concentration modulates the carbon flux direction. Similarly, Jeung *et al*. used acetate as the sole carbon source for MVA production. After addressing precursor supply and metabolic flux issues, they optimized the fermentation process *via* repeated fed-batch fermentation: acetate was continuously supplied to maintain a concentration of 5–10 g/L, avoiding acetate toxicity. After 156 h of fermentation, the final MVA titer reached 7.9 g/L, which is the highest titer reported for acetate-based MVA production [64].

## 7 Downstream processes for the lactonization of MVA to MVAL

The conversion of microbially fermented MVA to MVAL *via* low-pH heating-induced cyclization is currently recognized as the mainstream technical route for MVAL production in synthetic biology. As highlighted earlier, most studies have focused exclusively on improving MVA fermentation titers, with very few systematic investigations of the esterification-cyclization step that converts MVA to MVAL. Notably, the quantification of MVA in many previous studies relied on an indirect method: first, MVA was converted to MVAL, followed by gas chromatography-mass spectrometry analysis of MVAL. Although minor variations exist across reports, the general protocol can be summarized as follows. The cell-free supernatant of the MVA-containing fermentation broth is acidified (often with prolonged heating) to facilitate MVA cyclization to MVAL. After saturation with anhydrous sodium sulfate, MVAL is extracted with ethyl acetate [23,65]. Importantly, this approach is intended solely for MVA quantification and not for large-scale MVAL production. It is reasonable to speculate that ADEKA converted the MVA produced by *S. fibuligera* into MVAL *via* downstream acidification and heating-induced cyclization, although, to our knowledge, detailed technical information from ADEKA has not been disclosed. The only explicitly reported case of systematic research on the MVA-to-MVAL conversion step is described in [9]. After obtaining a high-titer MVA fermentation broth *via* upstream processes, the first step involves decolorization. Activated carbon of different types (granular *vs*. powdered) and mesh sizes (4–8 mesh, 20–60 mesh, and 100 mesh) were tested: 5% (w/v) activated carbon was added to the cell-free fermentation broth, and both decolorization efficiency and the MVA recovery rate were evaluated. Powdered activated carbon (20–60 mesh and 100 mesh) achieved complete decolorization (resulting in a colorless, transparent broth) with an MVA recovery rate >98%, minimizing product loss. Among these, 20–60 mesh powdered activated carbon offered the best cost-effectiveness. While 100 mesh activated carbon exhibited comparable decolorization performance, its slow filtration rate made it unsuitable for scale-up. MVA spontaneously lactonizes to MVAL under acidic conditions, and the key challenge is selecting a catalyst that enables a high conversion efficiency without generating byproducts. Traditionally, sulfuric acid has been used, but it produces the unwanted byproduct anhydro-MVAL [24,54,86,87]. In this study, phosphoric acid was tested for the first time, and its performance was compared with that of sulfuric acid. The results showed that phosphoric acid was the optimal catalyst: at a 1:5 molar ratio (phosphoric acid to MVA), a conversion rate of 90.4% was achieved without any byproducts, resolving the byproduct issue associated with sulfuric acid. The main conversion process was completed within 9 h, making it suitable for continuous industrial production. Nuclear magnetic resonance analysis confirmed that the biosynthesized MVAL exclusively corresponded to the (*R*)-enantiomer with a chiral purity of >99%.

## 8 Perspectives

The microbial synthesis of MVAL has achieved remarkable progress in chassis optimization, enzyme engineering, and fermentation regulation, with MVA titers reaching industrially relevant levels (Table 1). To advance the transition from laboratory research to large-scale production, future research must overcome the limitations of a single technology and realize the cross-disciplinary integration of synthetic biology, enzyme engineering, process engineering, and artificial intelligence (AI). *S. fibuligera* strains, pioneered by ADEKA for industrial MVA production, have a notable limitation in that there is no established genetic manipulation system. This limitation prevents rational engineering from further unlocking the potential of MVA synthesis. Regrettably, these proprietary strains are not commercially available to the broader research community. Nevertheless, widely accessible high-performance chassis such as *S. cerevisiae, Y. lipolytica*, and *E. coli* have been validated for high MVA production, offering viable alternatives for academic and industrial research.

For pathway optimization, the spatial organization of the MVA pathway enzymes has been explored *via* protein- or RNA-based scaffolds to enhance intermediate channeling. However, a more conventional strategy, namely, the development of multifunctional fusion enzymes (e.g., fusing ACS and AACoAT *via* flexible linkers), remains underexplored. This approach could form efficient metabolic channels to minimize intermediate diffusion loss and improve flux toward MVA. Additionally, the efficiency of key MVA pathway enzymes (AACoAT, HMGS, and HMGR) directly determines the product titer. However, current engineering strategies, such as single-point mutations and RBS optimization at the translational level, only yield incremental improvements. Future enzyme engineering studies should leverage AI-driven rational design combined with high-throughput screening [88].

Traditional metabolic regulation strategies, such as constitutive promoter-mediated overexpression and static gene knockout, often trigger metabolic network imbalances or cell growth inhibition [89]. In contrast, dynamic feedback control and multi-omics-guided global reprogramming are emerging as core directions for future metabolic engineering. Metabolite-responsive genetic circuits can be constructed to achieve the on-demand distribution of metabolic flux. For instance, acetyl-CoA biosensors based on the transcription factors AccR [90] or BkdR [91] and NADPH biosensors [92] tailored to regulate PntAB expression can autonomously balance the precursor and cofactor supply without manual parameter adjustment, thus overcoming the limitations of static regulation. Furthermore, integrating transcriptomics, metabolomics, and proteomics datasets [93] and efficiently deciphering the datasets *via* AI technologies, such as machine learning and deep learning, enables the identification of cryptic metabolic bottlenecks [88]. By leveraging these AI-identified insights, CRISPR-based multiplex genome editing can be deployed to optimize global metabolic flux, thereby further boosting the precision and efficiency of MVA biosynthesis.

Building on the two-step microbial MVAL synthesis route discussed above, a logical question arises: can one-step microbial production of MVAL be achieved directly? Unfortunately, this approach faces substantial bottlenecks that render it nearly unfeasible with inherent economic disadvantages, even if it is technically achievable. Intramolecular cyclization of MVA to MVAL typically occurs under acidic and high-temperature conditions in organic solvents (e.g. as ethyl acetate). For *in* s*itu* conversion during fermentation, the culture broth requires a sustained high temperature and low pH, imposing stringent acid and thermotolerance demands on the chassis. Conventional hosts, such as *E. coli*, are incompatible with these extreme conditions. Moreover, maintaining high temperatures throughout the fermentation process results in excessive energy consumption, thereby undermining economic viability. An alternative strategy involves mining and expressing esterase-encoding genes to catalyze MVA lactonization. However, no esterases with suitable performance for direct application in this context have been reported, necessitating extensive mining and screening efforts. Most importantly, MVAL is highly susceptible to hydrolysis in aqueous environments. Regardless of the conversion method, liquid fermentation media contain large amounts of water, leading to the rapid ring-opening of MVAL and its reversion to MVA, even if *in situ* synthesis is achieved. Thus, the current efforts are focused on optimizing the established two-step process (MVA synthesis followed by chemical lactonization) *via* the chassis, pathway, and process engineering strategies outlined above.

From an industrial perspective, high raw material costs remain a major challenge. Replacing glucose with low-cost lignocellulosic hydrolysates (e.g., corn stover and wheat straw) is a critical strategy for reducing production costs [94]. Although studies have explored cheap carbon sources (e.g., 2,3-BDO, cellobiose, and acetate) for *E. coli* and *P. putida*, these efforts are insufficient for large-scale applications. Industrial fermentation feedstocks should adhere to the principles of “local availability,” “low cost,” and “sustainability,” prioritizing agricultural byproducts, food processing waste, and other low-value raw materials over refined sugars or chemically synthesized substrates. Ideally, lignocellulosic hydrolysates could serve as the main carbon source supplemented with cheap acetate, given its ability to be rapidly converted to acetyl-CoA, the key precursor for MVA.

In conclusion, advancements in microbial MVAL production will depend on interdisciplinary integration. With breakthroughs in chassis diversification, intelligent enzyme evolution, and dynamic metabolic regulation, microbial synthesis will eventually replace chemical synthesis as the mainstream method of MVAL production. This transition will not only meet the growing demand for high-chiral-purity MVAL in industries such as cosmetics but also promote the sustainable development of biomanufacturing, providing a new paradigm for the production of high-value chiral compounds.

## CRediT authorship contribution statement

**Hao Tang:** Writing – original draft, Visualization, Conceptualization. **Lihong Yin:** Writing – original draft. **Yiwen Jiang:** Writing – review & editing, Conceptualization. **Teng Xu:** Writing – review & editing. **Ting Xue:** Writing – review & editing. **Meimei Fu:** Writing – review & editing. **Jianghao Chen:** Writing – review & editing. **Jinshan Guo:** Writing – review & editing, Writing – original draft.

## Declaration of competing interest

The authors declare that they have no known competing financial interests or personal relationships that could have appeared to influence the work reported in this paper.
